# Sequencing reveals protective and pathogenic effects on development of diabetes of rare *GLIS3 *variants

**DOI:** 10.1371/journal.pone.0220805

**Published:** 2019-08-15

**Authors:** Jihua Sun, Christian Theil Have, Mette Hollensted, Niels Grarup, Allan Linneberg, Oluf Pedersen, Jens Steen Nielsen, Jørgen Rungby, Cramer Christensen, Ivan Brandslund, Karsten Kristiansen, Wang Jun, Torben Hansen, Anette P. Gjesing

**Affiliations:** 1 Biology Department, University of Copenhagen, Copenhagen, Denmark; 2 BGI-Europe, Copenhagen, Denmark; 3 Novo Nordisk Foundation Center for Basic Metabolic Research, Faculty of Health and Medical Sciences, University of Copenhagen, Copenhagen, Denmark; 4 Center for Clinical Research and Prevention, Bispebjerg and Frederiksberg Hospital, Copenhagen, Denmark; 5 Department of Clinical Experimental Research, Rigshospitalet, Glostrup, Denmark; 6 Department of Clinical Medicine, Faculty of Health and Medical Sciences, University of Copenhagen, Copenhagen, Denmark; 7 DD2, Steno Diabetes Center Odense, Odense University Hospital, Odense, Denmark; 8 Bispebjerg Hospital, University of Copenhagen, Denmark Laboratory of Genomics and; 9 Department of Internal Medicine and Endocrinology, SLB, Hospital Lillebaelt, Vejle, Denmark; 10 Department of Clinical Biochemistry, Hospital Lillebaelt, Vejle, Denmark; 11 Institute of Regional Health Research, University of Southern Denmark, Odense, Denmark; 12 Laboratory of Genomics and Molecular Biomedicine, Department of Biology, University of Copenhagen, Copenhagen, Denmark; 13 BGI-Research, Shenzhen, China; 14 iCarbonX, Shenzhen, China; Children's hospital of philadelphia, UNITED STATES

## Abstract

**Background:**

Based on the association of common *GLIS3* variants with various forms of diabetes and the biological role of GLIS3 in beta-cells, we sequenced *GLIS3* in non-diabetic and diabetic Danes to investigate the effect of rare missense variants on glucose metabolism.

**Methods:**

We sequenced 53 patients with maturity-onset diabetes of the young (MODY), 5,726 non-diabetic participants, 2,930 patients with newly diagnosed type 2 diabetes and 206 patients with glutamic acid decarboxylase antibody (GADA) -positive diabetes.

**Results:**

In total we identified 86 rare (minor allele frequency < 0.1%) missense variants. None was considered causal for the presence of MODY. Among patients with type 2 diabetes, we observed a higher prevalence of rare *GLIS3* missense variants (2.5%) compared to non-diabetic individuals (1.8%) (odds ratio of 1.37 (interquartile range:1.01–1.88, *p* = 0.04)). A significantly increased HbA1c was found among patients with type 2 diabetes and with GADA-positive diabetes carrying rare *GLIS3* variants compared to non-carriers of rare *GLIS3* variants with diabetes (*p* = 0.02 and *p* = 0.004, respectively). One variant (p.I28V) was found to have a minor allele frequency of only 0.03% among patients with type 2 diabetes compared to 0.2% among non-diabetic individuals suggesting a protective function (odds ratio of 0.20 (interquartile range: 0.005–1.4, *p* = 0.1)), an effect which was supported by publically available data. This variant was also associated with a lower level of fasting plasma glucose among non-diabetic individuals (*p* = 0.046).

**Conclusion:**

Rare missense variants in *GLIS3* associates nominally with increased level of HbA1c and increased risk of developing type 2 diabetes. In contrast, the rare p.I28V variant associate with reduced level of fasting plasma glucose and may be protective against type 2 diabetes.

## Introduction

*GLIS3* is encoding a member of the Krüppel-like zinc finger protein subfamily GLI-similar 3 which is a transcription factor playing a critical role both as a repressor and activator of transcription [1]. Human GLIS3 exist in two isoforms: Isoform A which is the longest including a total of 930 amino acids, and isoform B which is 155 amino acids shorter at the N-terminus [2]. Isoform A is highly expressed in the pancreas, kidney, and thyroid, whereas the smaller isoform B is strongly expressed in the heart, liver, kidney, and skeletal muscle [3]. The transcriptional regulation by GLIS3 is mediated through an interaction between GLIS-binding sites (GLISBS) in the regulatory region of target genes and zinc finger domains in GLIS3 [1, 4].

Globally *Glis3* deficient mice have a decreased beta-cell mass and develop neonatal diabetes in addition to hypothyroidism, and cystic kidney disease [5]. In addition, Glis3 missense variants identified in Goto-Kakizaki rats have been found to increase basal production of insulin but reduce the glucose stimulated insulin secretion in INS1-cells [6].

Also in humans, mutations in *GLIS3* are a rare cause of neonatal diabetes as well as congenital hypothyroidism and in some cases in combination with polycystic kidney disease, glaucoma, and hepatic fibrosis in a recessive manner [3, 7]. It has been found for the neonatal diabetes genes *GCK* and *PDX1*, that homozygous mutations are a cause of neonatal diabetes and heterozygote mutations results in the monogenic form of diabetes called maturity onset diabetes of the young (MODY) [8, 9]. This may also be the case for *GLIS3*.

Type 1 diabetes (T1D) and T2D are two genetically distinct diseases, yet *GLIS3* is one of the few genes in which common variants have been found to associate with both forms of diabetes [10, 11]. In addition, common *GLIS3* variants associate with development of gestational diabetes [12], with increased level of fasting glucose [10] and with altered insulin clearance [13]. Thus, *GLIS3* appear to be implicated in various diabetes forms.

This pathogenic effect of *GLIS3* variants may be related to GLIS3 being instrumental for an optimal transcriptional activation of Ins2 in conjunction with the transcriptions factors Pdx1, NeuroD1, and MafA [14]. This has been further verified by the observation that mutations in the GLISBS of the insulin promoter are responsible for the development of neonatal diabetes in some patients [14]. However, GLIS3 has also been shown in INS1-cells to stimulate the transcription of genes of importance for apoptosis such as Atg7 and Atg4a [6] as well as the pro-apoptotic BH3-only protein Bim [15].

In humans common and low frequency *GLIS3* variants (MAF > 0.1%) have previously been investigated in relation to diabetes [10]. Thus, we sequenced the coding region of *GLIS3* using next-generation sequencing (NGS) in order to investigate the effect of rare *GLIS3* missense variants in a well characterised Danish population. The sequencing was completed in 53 MODY patients with an unknown genetic etiology (MODYX), 5,726 non-diabetic individuals, 2,930 patients newly diagnosed with T2D and 206 patients with GADA-positive diabetes with the aim to investigate 1) if *GLIS3* missense variants is involved in MODYX, 2) if rare *GLIS3* missense variants increase the risk of T2D development and 3) if rare *GLIS3* missense variants affect measures of glucose metabolism.

## Materials and methods

### Population

Individuals in the present study include: 1) 53 MODYX probands recruited from the outpatient clinic at Steno Diabetes Center, Copenhagen, Denmark, 2) 5,726 non-diabetic individuals recruited from the Danish population-based Inter99 study [16, 17] including 1,157 prediabetic individuals (having impaired fasting glucose (IFG) and/or impaired glucose tolerance (IGT)) and 4,569 glucose tolerant individuals (NGT) classified according the WHO 1999 criteria using a 2-hour oral glucose tolerance test (OGTT) [18]; 3) 2,930 patients with newly-diagnosed T2D recruited through the DD2-cohort [19]; and 4) 206 patients with GADA-positive diabetes recruited from the DD2-cohort (n = 85) and at Hospital Lillebaelt, Denmark (n = 121). A clinical description of included individuals can be found in Table 1.

**Table 1:** Clinical description of participants.

Trait	Glucose tolerant (Inter99, *n* = 4,569)	Pre-diabetes (Inter99, *n* = 1,157)	T2D patients (*n* = 2,930)	GADA-positive diabetes patients (*n* = 206)	MODYX probands (*n* = 53)
**Sex (*n*)**	2,113/2,456	700/457	1,678/1,177	108/95	25/ 28
**Age (mean, SD)**	45.2 (7.87)	48.8 (7.33)	60.2 (11.1)	49.3 (12.6)	22.4 (14.1)
**BMI (kg/m**^**2**^**)**	25.5 (4.14)	28.1 (4.97)	31.4 (6.29)	27.2 (5.67)	23.72 (6.16)
**Fasting plasma glucose (mmol/l)**	5.31 (0.40)	6.00 (0.52)	7.49 (1.73)	10.0 (4.41)	8.47 (2.94)
**Fasting serum- C-peptide (pmol/l)**	542.1 (217.2)	731.9 (315.3)	1243 (551.6)	433 (607.1)	503.8 (287.1)
**Fasting serum triglyceride (mmol/L)**	1.18 (0.94)	1.71 (2.19)	1.98 (1.43)	1.17 (0.69)	1.75 (0.75)
**Fasting serum total cholesterol (mmol/L)**	5.43 (1.03)	5.83 (1.14)	4.52 (1.08)	4.50 (0.90)	4.81 (0.78)

Data presented as mean (SD).

Patients with newly diagnosed T2D were GADA-negative and had a fasting serum C-peptide concentration > 150 pmol/l within 1.5 years from diabetes diagnosis if C-peptide was available. GADA-positive patients were selected based on a GADA>30 IU/ml.

Prior to participation, written informed consents were obtained from all participants. The study design was in accordance with the ethical scientific principles of the Helsinki Declaration II and approved by The Scientific Ethics Committee of the Capital Region of Denmark (Inter99: KA-98155) and by the Danish National Ethical Committee on Health Research (DD2: S-20100082).

### Anthropometrics measurements

Body weight, height, waist, and hip circumference were measured with light indoor clothes and without shoes. Body mass index (BMI) was defined as body mass divided by body height in units of kg/m^2^.

### Metabolic measurements

#### Inter99 participants and participants recruited at Hospital Lillebaelt, Denmark

The blood samples were drawn after a 12h fast for measures of glycated hemoglobin (HbA1c), plasma glucose, serum insulin, serum C-peptide, total cholesterol and serum triglyceride [16, 20]. In addition, individuals without known diabetes underwent a standard 75g OGTT with samples drawn at 30, and 120 minutes measured for plasma glucose, serum insulin and C-peptide. Serum insulin levels (excluding des-31,32 and intact proinsulin) were measured using the AutoDELFIA insulin kit (Perkin-Elmer, Wallac, Turku, Finland). Plasma glucose was analysed using a glucose oxidase method (Granutest; Merck, Darmstadt, Germany)[17]. Concentrations of serum triglycerides and total cholesterol were analysed using enzymatic colorimetric methods (GPO-PAP and CHOD-PAP, Roche Molecular Biochemicals, Germany). HbA1c was measured using ion-exchange high performance liquid chromatography (normal reference range: 4.1–6.4%) [21].

#### Participants from the DD2-cohort

Measures of BMI, and routine laboratory measurements such as fasting plasma glucose, fasting serum C-peptide and GADA were extracted from the Danish Diabetes Database for Adults [22].

### Estimates of glucose metabolism

Oral glucose-stimulated insulin response was measured as the insulinogenic index. Homeostatic model assessment of insulin resistance (HOMA-IR) index measured as previous reported [23].

### Targeted resequencing

A customized oligonucleotide probe was designed including all coding regions of *GLIS3* as previously described [24]. Genomic DNA was extracted from human leucocyte nuclei. Target regions were captured and subsequently underwent library construction. The captured DNA libraries were sequenced using the Illumina HiSeq2000 as paired-end 90 bp reads (following the manufacturer’s standard cluster generation and sequencing protocols). *GLIS3* was covered with a minimum depth of over 20X and a mean depth of the target region of 166X. Qualified reads were aligned to the reference of human genome (UCSC hg19) using the Burrows-Wheeler Aligner tool (http://bio-bwa.sourceforge.net), and single-nucleotide polymorphisms and indels were identified using the Genome Analysis Toolkit (https://www.broadinstitute.org/gatk/). The variants were annotated using Annovar [25] with variants annotated according to transcript NM_001042413.

The linkage disequilibrium (LD) structure between presently identified and previously investigated variants was calculated using LDlink [26].

### Statistical analysis

The statistical differences in carrier-frequency between cases and controls were calculated using chi-squared, a kernel-based adaptive cluster (KBAC) test as well as an analysis which collapses and combines rare variants (CMC) [27]. The CMC analysis included adjustment for principal component 1–4 in order to adjust for population stratification.

However, due to the low number of carriers of each group, the statistical difference between cases and control for variant p.I28V was calculated using a fisher’s exact test. Quantitative trait analyses were performed using linear regression using additive genetic models with adjustment for age and sex. Traits were all q-transformed prior to analysis. Statistical analyses were performed using RStudio software version R-3.4.1 (version 3.2.3; R Foundation for Statistical Computing, Boston, MA, USA) except KBAC and CMC which was performed using rvtests [28]. Burden-test for the association between variants in *GLIS3* and glucose tolerance status did not undergo correction for multiple testing as this was a test of our primary hypothesis and a *p*-value < 0.05 was considered statistically significant. In contrast, quantitative analyses were corrected for multiple testing. Thus, for quantitative traits the adjusted level of significance is p > 0.006 (0.05/9).

### Estimation of functionality

The pathogenicity of missense variants was evaluated using the Combined Annotation Dependent Depletion (CADD) score where a PHRED-scaled CADD score above 10 predicts pathogenicity in the top 10 percentile of all variants and a score above 20 predicts the top 1 percentile [29].

## Results

The coding regions of *GLIS3* were sequenced in 53 MODYX patients, 2,930 patients with T2D, 5,724 non-diabetic individuals and 206 patients with GADA-positive diabetes. A total 88 missense variants were identified of which three were common variants (MAF >1%), six were low frequency variants (MAF between 1% and 0.1%) and the vast majority (n = 79) were rare (MAF<0.1%) (S1 Table).

Three variants were found among the patients having MODYX. These included the common variants p.G313A and p.D512E and the rare variant p.V916L. However, the rare variant p.V916L was also found among three patients with T2D and five glucose tolerant individuals (Table 1), thus, none of these variants can be considered causal for the development of MODY.

Among the remaining patients with T2D and participants without diabetes, we found three common variants (p.G313A, p.P456Q, p.D512E) and six low frequency variants (p.P282A, p.S298Y, p.P364S, p.Q397H, p.H400R and p.E515D) which have previously been investigated in a large GWAS study combining GWAS data from close to 900,000 individuals [30]. Thus, we did not further investigate the effect of neither common nor low frequency variants but focussed on the previously un-investigated effect of rare variants in *GLIS3*.

A total of 79 rare variants were found among 100 non-diabetic individuals including three individuals carrying two variants and among 70 patients with T2D of whom two individuals are carrying two variants. Thus, the prevalence of rare *GLIS3* variants was 2.4% among patients with T2D compared to 1.7% among non-diabetic individuals revealing a slight enrichment of rare *GLIS3* variants in patients having T2D with odds ratio (OR) of 1.37 (IQR: 1.01–1.85, *p* = 0.04, Table 2).

**Table 2:** The prevalence of rare (MAF<0.1%) *GLIS3* missense mutations among non-diabetic individuals (IGT+IFG), patients with T2D.

	**Non-diabetic (*n* = 5,726)**	**T2D patients** **(*n* = 2,930)**	**Non-diabetic versus T2D patients:**		
**Carriers of *GLIS3* variants**	100	70	**Fishers exact** OR (95% CI), *p*-value	**Kbac**	**CMC**
**Non-carriers of *GLIS3* variants**	5,626	2,860	1.37 (1.01–1.88) *p* = 0.04	*p* = 0.02	*p* = 0.04
	**Non-diabetic (*n* = 5,726)**	**T2D** **(*n* = 2,930)**	**Non-diabetic versus T2D patients:**		
**Carriers of *GLIS3* variants excluding p.I28V**	90	69	**Fishers exact** OR (95% CI), *p*-value	**Kbac** *p*-value	**CMC** *p*-value
**Non-carriers of *GLIS3* variants**	5,626	2,860	1.51 (1.08–2.09) p = 0.01	*p* = 0.01	*p* = 0.01

OR (Odds ratios) and *p*-values are adjusted for age and sex. CI: 95% confidence interval.

The effect of *GLIS3* mutations on glucose metabolism was further explored and in patients with T2D, levels of HbA1c were nominally elevated to 7.20% (SD:1.69) among carriers of rare *GLIS3* variants compared to 6.80% (SD:1.23) among non-carriers, *p* = 0.02 (Table 3). As variants in *GLIS3* also have been found to associate with T1D, we investigated the effect of rare *GLIS3* variants in among patients classified as T2D, yet being GADA-positive (n = 206). Three carriers of *GLIS3* variants were found with two carriers of the p.P96L and one carrier of p.P703S (S1 Table). Among these, a significantly higher level of HbA1c (11.37%, SD: 2.99) compared to non-carriers (7.62%, SD: 1.40), *p* = 0.004 was also found (Table 3).

**Table 3:** Quantitative analysis of rare *GLIS3* variants in 5,726 non-diabetic individuals, 2,930 patients with T2D and 206 patients with GADA-positive diabetes.

Trait	Non-carriers	Carriers	*p*-value
**Non-diabetic individuals (*n* = 5,726)**			
*n* (men/women)	**2,763/2,863**	**50/50**	NA
Age (years)	45.9 (7.61)	47.5 (7.11)	0.1
BMI (kg/m^2^)	26.0 (4.43)	26.5 (4.75)	0.5
Waist/hip ratio	0.85 (0.09)	0.86 (0.09)	0.6
Glycated hemoglobin (HbA1c %)	5.79 (0.40)	5.83 (0.42)	0.7
Glycated hemoglobin (HbA1c mmol/mol)	39.8 (4.35)	40.3 (4.61)	0.7
HOMA-IR	1.67 (1.14)	1.83 (1.22)	0.1
Fasting plasma glucose (mmol/l)	5.45 (0.51)	5.47 (0.51)	0.9
30-min plasma glucose (mmol/l)	8.56 (1.70)	8.60 (1.78)	0.8
2-h plasma glucose (mmol/l)	5.94 (1.53)	5.90(1.45)	0.7
0-min serum C-peptide (pmol/l.min)	579.8 (251.1)	616.6 (302.3)	0.3
30-min serum C-peptide (pmol/l.min)	2001 (712.0)	2076 (884.6)	0.5
2-h serum C-peptide (pmol/l.min)	2253 (975.5)	2311 (1030)	0.7
Fasting serum insulin (pmol/l)	40.8 (26.4)	44.3 (27.6)	0.5
30-min serum insulin (pmol/l)	290.6 (180.9)	302.5 (215.7)	0.09
2-h serum insulin (pmol/l)	203.8 (193.1)	226.2 (248.9)	0.5
Insulinogenic Index	29.7 (19.3)	30.8 (22.3)	0.5
**T2D patients**			
n (men/women)	1638/1151	40/26	NA
Age (years)	60.7 (11.6)	60.2 (10.2)	0.5
BMI (kg/m^2^)	31.4 (6.29)	31.1 (5.52)	0.9
W/H ratio	0.98 (0.094)	0.97 (0.076)	0.6
Glycated hemoglobin (HbA1c %)	6.80 (1.23)	7.20 (1.66)	**0.02**
Glycated hemoglobin (HbA1c mmol/mol)	50.8 (13.5)	55.2 (18.1)	**0.02**
Onset (years)	59.3 (11.65)	59.1 (10.2)	0.9
Fasting plasma glucose (mmol/l)	7.49 (1.74)	7.24 (1.57)	0.3
Fasting serum C-peptide (pmol/l.min)	1246 (553.6)	1116 (441.7)	0.1
**GADA-positive diabetes patients**			
n (men/women)	106/94	2/1	NA
Age (years)	47.2 (11.8)	31.9 (4.37)	0.1
BMI (kg/m^2^)	27.2 (5.66)	24.4 (6.71)	0.3
W/H ratio	0.92 (0.082)	0.87 (0.097)	0.3
Glycated hemoglobin (HbA1c %)	7.62 (1.40)	11.37 (2.99)	**0.004**
Glycated hemoglobin (mmol/mol)	59.8 (15.3)	100.8 (32.7)	**0.004**
Onset (years)	36.4 (16.8)	29.7 (9.72)	0.4
Fasting plasma glucose (mmol/l)	9.88 (4.36)	16.7 (1.05)	0.07
Fasting serum C-peptide (pmol/l.min)	436.2 (610.9)	201.7 (177.3)	0.7

Data presented as mean (SD). *P*-values are adjusted for sex and age.

In contrast to the overall prevalence of rare variants, one of the identified rare variants (p.I28V) was far more frequent among non-diabetic individuals compared to patients with T2D with ten carriers among non-diabetic individuals and only one carrier with T2D. These non-diabetic carriers had a slightly lower level of fasting plasma glucose compared to non-diabetic individuals not carrying this variant (*p* = 0.047, S2 Table). The prevalence of this variant was further investigated in a larger publically available dataset where we observed an excess of five carriers among non-diabetic individuals (n = 9,335) in contrast to only one carriers among patients with T2D (n = 9,121) [31] which in a meta-analysis combined with our data disclose a combined OR of 0.20 (IQR: 0.046–0.90), *p* = 0.04. The publically available online data also identifies a significantly lower level of fasting glucose among carriers of this variant (beta = -0.21, *p* = 0.02) [31], an analysis which is performed in more than 75,000 individuals.

## Discussion

We investigated the effect of rare missense variants within *GLIS3* on risk of T2D and on measures of glucose homeostasis. Since amino acids 1–156 are only present in isoform A, 17 of the identified variants are only affecting this longer transcript. However, the longer isoform A is expressed in the pancreas, thus, we assume that all of the identified variants may have potential effect on glucose metabolism.

A slightly elevated prevalence of rare *GLIS3* missense variants was observed among patients with T2D compared to non-diabetic individuals. Supporting this observation, levels of HbA1c were also elevated among patients with T2D carrying rare *GLIS3* missense variants compared to patients not carrying such variants.

Despite the known function of GLIS3 in beta-cell function, we did not observe any effect of *GLIS3* mutations neither on circulating levels of C-peptide nor levels of insulin. Thus, we were unable to determine the cause of elevated HbA1c measuring the long-term glycaemic control. Our observations are well in line with GWAS, finding that common *GLIS3* variants are associated with T2D and elevated levels of fasting glucose, however, no other measures of glucose homeostasis have been found to be affected by common *GLIS3* variants.

It is puzzling that *GLIS3* variants, do not appear to affect insulin secretion in human studies as GLIS3 has been found to be: 1) directly implemented in the transcription of INS [14], 2) been found to be involved in pancreas maturation [7], 3) been found to be involved in beta-cell apoptosis [32] and 4) the physiological consequence of *GLIS3* mutations results in diabetes. Yet, direct effects of the mutations on the protein and on beta-cell function are still undisclosed.

One of the identified variants (p.I28V) was less prevalent among patients with T2D and associated with reduced levels of fasting plasma glucose both in our data and in online available data. This suggests a protective effect of this variant on the development of hyperglycaemia. This potential protective variant is only present in Europeans (MAF = 0.1%) and to a smaller extend in the Hispanic population (MAF = 0.02%), indicating that this is mutation is a *de novo* mutations arisen in Europeans. In a Japanese study, a *GLIS3* variant (p.A908V) protecting against T1D was found among approximately 3,000 Japanese patients with T1D and control individuals [33], indicating that variants in *GLIS3* may not only have a deleterious effects on diabetes.

Thus, there appear to be large differences in the pathogenicity of variants within *GLIS3*. Within the current population, we do not encounter variants having a highly detriment effect leading to the development of monogenic diabetes. The combined effect of rare missense variant only results in a modest increased risk of T2D and only a slight increase in level of HbA1c. In contrast, a possible protective variant was encountered. The effect of the identified *GLIS3* mutations must be further investigated in functionality studies of each encountered variant.

Due to the central role of GLIS3 in glucose metabolism, GLIS3 may be considered a potential treatment target and two studies have found compounds able to significantly diminish the apoptotic effect of GLIS3. These compounds may be developed further into anti-diabetic treatments in the future [15, 32].

In conclusion, we find that rare missense variants in *GLIS3* may increase risk of diabetes and elevate levels of plasma glucose. However, we also find a rare missense variant which likely protects against the development of diabetes. Thus, variants in *GLIS3* may explain a fraction of heterogeneity in susceptibility to T2D.

## Supporting information

S1 Table Identified *GLIS3* missense variants among non-diabetic individuals, patients with T2D and patients with GADA-positive diabetes. (DOCX)

S2 Table The effect of the p.I28V variants among non-diabetic participants. (DOCX)

## Abbreviations

BMI: body mass index
CI: confidence interval
GADA: glutamic acid decarboxylase antibody
GDM: gestational diabetes mellitus
hsCRP: high-sensitivity CRP
IFG: impaired fasting glycaemia
IGT: impaired glucose tolerance
IQR: Interquartile range
MAF: minor allele frequency
MODY: maturity-onset diabetes of the young
OHA: oral hyperglycaemic agent
OR: Odds ratio
T2D: type 2 diabetes
