# Supplementary material for: Sequencing reveals protective and pathogenic effects on development of diabetes of rare GLIS3 variants
Source: PLoS One. 2019 Aug 15;14(8):e0220805. doi: 10.1371/journal.pone.0220805 (PMC6695102; doi:10.1371/journal.pone.0220805)
Supplement: S1 Table — (DOCX) [file pone.0220805.s001.docx]

**S1 table:** Identified *GLIS3* missense variants among non-diabetic individuals, patients with T2D and patients with GADA-positive diabetes.

| **Variants** | **Rs-number** | **Position** | **MAF Gnomad**  **(All)** | **CADD-score** | **MAF our cohort** | **T2D patients (het/ho)** | **Non-diabetic**  **(het/ho)** | **GADA-positive**  **(het/ho)** | **MODYX**  **(het/ho)** |
| --- | --- | --- | --- | --- | --- | --- | --- | --- | --- |
| **Common variants MAF > 1%** | | | | | | | | | |
| p.G313A | rs35154632 | 4118540 | 0.0081 | 25.6 | 0.01 | 69/1 | 114/0 | 3/0 | 2/0 |
| p.P456Q | rs6415788 | 4118111 | 0.66 | 0.622 | 0.57 | 1381/493 | 2744/ 995 | 102/27 | 35/0 |
| p.D512E | rs148199056 | 4117942 | 0.017 | 12.13 | 0.021 | 126/1 | 224/7 | 11/0 | 2/0 |
| **Low frequency variants 1% < MAF > 0.1%** | | | | | | | | | |
| p.P282A | rs143051164 | 4118634 | 2.0*10^-3^ | 8.40 | 4.0*10^-3^ | 18/0 | 47/0 | 2/0 | 0/0 |
| p.S298Y | rs148572278 | 4118585 | 2.0*10^-3^ | 25.6 | 2.0*10^-3^ | 11/0 | 23/0 | 1/0 | 0/0 |
| p.P364S | rs143056249 | 4118388 | 1.4*10^-3^ | 0.001 | 1.0*10^-3^ | 5/0 | 15/0 | 1/0 | 0/0 |
| p.Q397H | rs138497710 | 4118287 | 2.1*10^-3^ | 20.5 | 4.0*10^-3^ | 22/0 | 46/0 | 0/0 | 0/0 |
| p.H400R | rs376031632 | 4118279 | 8.0*10^-4^ | 18.11 | 2.0*10^-3^ | 9/0 | 32/0 | 1/0 | 0/0 |
| p.E515D | rs72687988 | 4117933 | 2.9*10^-3^ | 24.2 | 2.0*10^-3^ | 9/0 | 23/0 | 1/0 | 0/0 |
| Total low frequency | | | | | | 74/0 | 186/0 | 0/0 |  |
| **Rare variants MAF < 0.1%** | | | | | | | | |  |
| p.C6G | rs767988875 | 4286410 | 1.8*10^-5^ | 19.3 | 1.3*10^-4^ | 1/0 | 2/0 | 0/0 | 0/0 |
| p.H11Y | rs773069755 | 4286395 | 1.1*10^-4^ | 22.3 | 4.4*10^-5^ | 0/0 | 1/0 | 0/0 | 0/0 |
| p.T13I | NA | 4286388 | - | 21.9 | 4.4*10^-5^ | 1/0 | 0/0 | 0/0 | 0/0 |
| p.S24N | NA | 4286355 | - | 17.6 | 4.4*10^-5^ | 0/0 | 1/0 | 0/0 | 0/0 |
| p.I28V | rs113754532 | 4286344 | 2.5*10^-4^ | 14.4 | 4.9*10^-4^ | 1/0 | 10/0 | 0/0 | 0/0 |
| p.R32Q | rs375834888 | 4286331 | 2.8*10^-5^ | 23.7 | 8.9*10^-5^ | 1/0 | 1/0 | 0/0 | 0/0 |
| p.G36R | rs199788224 | 4286320 | 1.2*10^-4^ | 30.0 | 2.2*10^-4^ | 2/0 | 2/0 | 0/0 | 0/0 |
| p.S53R | NA | 4286267 | 4.1*10^-6^ | 23.0 | 4.4*10^-5^ | 1/0 | 0/0 | 0/0 | 0/0 |
| p.L54F | NA | 4286266 | - | 13.3 | 4.4*10^-5^ | 0/0 | 1/0 | 0/0 | 0/0 |
| p.S77G | rs775442287 | 4286197 | 4.1*10^-6^ | 24.3 | 4.4*10^-5^ | 1/0 | 0/0 | 0/0 | 0/0 |
| p.R78H | rs200195201 | 4286193 | 2.9*10^-5^ | 19.2 | 2.2*10^-4^ | 1/0 | 2/0 | 0/0 | 0/0 |
| p.L84F | rs200986848 | 4286174 | 2.1*10^-4^ | 24.6 | 1.8*10^-4^ | 1/0 | 2/0 | 0/0 | 0/0 |
| p.P86A | rs368959854 | 4286170 | 2.4*10^-5^ | 15.2 | 2.2*10^-4^ | 1/0 | 4/0 | 0/0 | 0/0 |
| p.P96L | rs143425492 | 4286139 | 1.9*10^-4^ | 8.0 | 3.6*10^-4^ | 2/0 | 1/0 | 2/0 | 0/0 |
| p.S109P | rs753973362 | 4286101 | 2.2*10^-5^ | 4.9 | 4.4*10^-5^ | 1/0 | 0/0 | 0/0 | 0/0 |
| p.G120R | rs200701636 | 4286068 | 1.8*10^-5^ | 26.2 | 4.4*10^-5^ | 1/0 | 0/0 | 0/0 | 0/0 |
| p.I141T | rs202165554 | 4125908 | 2.2*10^-4^ | 25.4 | 5.3*10^-4^ | 3/0 | 8/0 | 0/0 | 0/0 |
| p.S165X | NA | 4125836 | - | 39 | 4.4*10^-5^ | 0/0 | 1/0 | 0/0 | 0/0 |
| p.Q170E | rs779077595 | 4125822 | 4.1*10^-5^ | 24.6 | 3.6*10^-4^ | 2/0 | 4/0 | 0/0 | 0/0 |
| p.A174S | NA | 4125810 | 3.2*10^-5^ | 23.8 | 4.4*10^-5^ | 0/0 | 1/0 | 0/0 | 0/0 |
| p.N187Y | NA | 4125771 | - | 25.7 | 4.4*10^-5^ | 0/0 | 1/0 | 0/0 | 0/0 |
| p.R232K | NA | 4118783 | - | 11.0 | 8.9*10^-5^ | 0/0 | 1/0 | 0/0 | 0/0 |
| p.V265F | rs143192828 | 4118685 | 2.8*10^-4^ | 19.6 | 4.4*10^-5^ | 0/0 | 1/0 | 0/0 | 0/0 |
| p.A329V | rs776646791 | 4118492 | 4.1*10^-6^ | 28.6 | 1.3*10^-4^ | 0/0 | 1/0 | 0/0 | 0/0 |
| p.I331L | NA | 4118487 | 4.1*10^-6^ | 16.4 | 4.4*10^-5^ | 1/0 | 0/0 | 0/0 | 0/0 |
| p.S357N | rs374752356 | 4118408 | 1.7*10^-5^ | 21.7 | 4.4*10^-5^ | 1/0 | 0/0 | 0/0 | 0/0 |
| p.P364R | rs772125440 | 4118387 | 7.6*10^-5^ | 14.8 | 1.3*10^-4^ | 0/0 | 1/0 | 0/0 | 0/0 |
| p.P364L | rs772125440 | 4118387 | 3.2*10^-5^ | 10.0 | 4.4*10^-5^ | 0/0 | 1/0 | 0/0 | 0/0 |
| p.L373V | rs200263979 | 4118361 | 1.2*10^-3^ | 6.1 | 4.9*10^-4^ | 3/0 | 7/0 | 0/0 | 0/0 |
| p.P376S | rs76342955 | 4118352 | 2.5*10^-3^ | 0.001 | 8.9*10^-5^ | 0/0 | 1/0 | 0/0 | 0/0 |
| p.G385D | rs200959196 | 4118324 | 2.0*10^-4^ | 0.003 | 4.4*10^-5^ | 1/0 | 0/0 | 0/0 | 0/0 |
| p.L398M | NA | 4118286 | - | 24.7 | 4.4*10^-5^ | 0/0 | 1/0 | 0/0 | 0/0 |
| p.P405L | rs764072714 | 4118264 | 5.9*10^-5^ | 11.9 | 4.4*10^-5^ | 0/0 | 1/0 | 0/0 | 0/0 |
| p.G406C | rs75462592 | 4118262 | 2.8*10^-3^ | 23.7 | 1.3*10^-4^ | 2/0 | 0/0 | 0/0 | 0/0 |
| p.N409K | rs534295783 | 4118251 | 7.2*10^-5^ | 14.8 | 4.4*10^-5^ | 1/0 | 0/0 | 0/0 | 0/0 |
| p.P420L | rs768040059 | 4118219 | 5.3*10^-6^ | 0.06 | 4.4*10^-5^ | 0/0 | 1/0 | 0/0 | 0/0 |
| p.T440A | rs80161424 | 4118160 | 1.0*10^-3^ | 0.001 | 4.4*10^-5^ | 1/0 | 0/0 | 0/0 | 0/0 |
| p.P445R | NA | 4118144 | NA | 0.01 | 1.8*10^-4^ | 3/0 | 1/0 | 0/0 | 0/0 |
| p.L473F | rs369088290 | 4118061 | 9.4*10^-5^ | 22.6 | 1.8*10^-4^ | 1/0 | 1/0 | 0/0 | 0/0 |
| p.P475L | rs769236310 | 4118054 | 1.4*10^-5^ | 0.003 | 4.4*10^-5^ | 0/0 | 1/0 | 0/0 | 0/0 |
| p.P475T | NA | 4118055 | - | 0.002 | 4.4*10^-5^ | 1/0 | 0/0 | 0/0 | 0/0 |
| p.D490V | rs371985224 | 4118009 | 1.1*10^-5^ | 24.0 | 4.4*10^-5^ | 1/0 | 0/0 | 0/0 | 0/0 |
| p.M493T | NA | 4118000 | 4.1*10^-6^ | 0.02 | 4.4*10^-5^ | 1/0 | 0/0 | 0/0 | 0/0 |
| p.I505V | rs369355792 | 4117965 | 1.6*10^-5^ | 15.8 | 1.3*10^-4^ | 1/0 | 0/0 | 0/0 | 0/0 |
| p.G540S | rs759183029 | 4117860 | 7.2*10^-6^ | 29.5 | 2.7*10^-4^ | 2/0 | 2/0 | 0/0 | 0/0 |
| p.A607T | NA | 3937081 | - | 32.0 | 2.2*10^-4^ | 2/0 | 2/0 | 0/0 | 0/0 |
| p.P626H | NA | 3932466 | - | 27.8 | 8.9*10^-5^ | 0/0 | 2/0 | 0/0 | 0/0 |
| p.R663W | NA | 3898832 | 8.1*10^-6^ | 35.0 | 4.4*10^-5^ | 0/0 | 1/0 | 0/0 | 0/0 |
| p.P684L | rs542599450 | 3898768 | 3.6*10^-5^ | 22.7 | 1.8*10^-4^ | 3/0 | 0/0 | 0/0 | 0/0 |
| p.A693T | rs568262538 | 3898742 | 2.5*10^-5^ | 9.3 | 3.1*10^-4^ | 3/0 | 4/0 | 0/0 | 0/0 |
| p.V697M | rs148816140 | 3898730 | 7.8*10^-4^ | 11.3 | 4.4*10^-5^ | 1/0 | 0/0 | 0/0 | 0/0 |
| p.R699H | rs149840771 | 3898723 | 2.5*10^-4^ | 15.8 | 2.2*10^-4^ | 0/0 | 3/0 | 0/0 | 0/0 |
| p.P703S | rs200705602 | 3898712 | 2.8*10^-5^ | 9.2 | 4.4*10^-5^ | 0/0 | 0/0 | 1/0 | 0/0 |
| p.I712N | NA | 3879589 | - | 23.4 | 4.4*10^-5^ | 0/0 | 1/0 | 0/0 | 0/0 |
| p.F713L | rs201347665 | 3879585 | 3.3*10^-5^ | 29.3 | 1.3*10^-4^ | 0/0 | 1/0 | 0/0 | 0/0 |
| p.S714C | rs139924264 | 3879583 | 1.4*10^-5^ | 18.7 | 4.4*10^-5^ | 1/0 | 0/0 | 0/0 | 0/0 |
| p.N716K | NA | 3879576 | 4.1*10^-6^ | 16.7 | 4.4*10^-5^ | 0/0 | 1/0 | 0/0 | 0/0 |
| p.N716S | rs756574107 | 3879577 | 8.1*10^-6^ | 6.9 | 2.7*10^-4^ | 3/0 | 2/0 | 0/0 | 0/0 |
| p.G726E | rs764370927 | 3879547 | 1.2*10^-5^ | 26.0 | 1.3*10^-4^ | 2/0 | 0/0 | 0/0 | 0/0 |
| p.Q753R | rs750346762 | 3879466 | 4.5*10^-5^ | 17.2 | 4.4*10^-5^ | 0/0 | 1/0 | 0/0 | 0/0 |
| p.H775Q | rs780948928 | 3856157 | 4.1*10^-6^ | 15.4 | 4.4*10^-5^ | 1/0 | 0/0 | 0/0 | 0/0 |
| p.R791T | NA | 3856110 | - | 23.2 | 4.4*10^-5^ | 1/0 | 0/0 | 0/0 | 0/0 |
| p.T792A | rs867985925 | 3856108 | 4.1*10^-6^ | 1.5 | 4.4*10^-5^ | 1/0 | 0/0 | 0/0 | 0/0 |
| p.H824Y | NA | 3856012 | - | 26.4 | 4.4*10^-5^ | 0/0 | 1/0 | 0/0 | 0/0 |
| p.K832E | rs779354091 | 3829472 | 1.5*10^-4^ | 23.2 | 4.4*10^-5^ | 1/0 | 0/0 | 0/0 | 0/0 |
| p.D840N | rs201704428 | 3829448 | 8.3*10^-5^ | 25.5 | 3.1*10^-4^ | 2/0 | 2/0 | 0/0 | 0/0 |
| p.I844T | rs193061752 | 3829435 | 4.3*10^-5^ | 0.003 | 1.8*10^-4^ | 2/0 | 2/0 | 0/0 | 0/0 |
| p.P846L | rs199505727 | 3829429 | 2.8*10^-4^ | 22.9 | 4.4*10^-5^ | 0/0 | 1/0 | 0/0 | 0/0 |
| p.D859G | NA | 3829390 | - | 25.2 | 4.4*10^-5^ | 1/0 | 0/0 | 0/0 | 0/0 |
| p.F871L | NA | 3829353 | - | 2.5 | 2.2*10^-4^ | 1/0 | 3/0 | 0/0 | 0/0 |
| p.S892C | rs749999750 | 3828391 | 8.2*10^-6^ | 16.1 | 4.4*10^-5^ | 0/0 | 1/0 | 0/0 | 0/0 |
| p.S894P | NA | 3828385 | 4.1*10^-6^ | 23.1 | 4.4*10^-5^ | 0/0 | 1/0 | 0/0 | 0/0 |
| p.L896F | rs76094493 | 3828379 | 1. 1*10^-2^ | 22.4 | 4.4*10^-4^ | 1/0 | 3/0 | 0/0 | 0/0 |
| p.F897C | NA | 3828375 | 8.2*10^-6^ | 25.2 | 4.4*10^-5^ | 1/0 | 0/0 | 0/0 | 0/0 |
| p.R902H | rs772126214 | 3828360 | 3.7*10^-5^ | 23.6 | 4.4*10^-5^ | 1/0 | 0/0 | 0/0 | 0/0 |
| p.G904R | rs150310830 | 3828355 | 2.1*10^-4^ | 24.6 | 4.4*10^-5^ | 0/0 | 1/0 | 0/0 | 0/0 |
| p.V916L | rs151140581 | 3828319 | - | 20.7 | 6.7*10^-4^ | 3/0 | 5/0 | 0/0 | 1/0 |
| p.V916M | rs151140581 | 3828319 | 1.4*10^-4^ | 23.6 | 8.9*10^-5^ | 1/0 | 1/0 | 0/0 | 0/0 |
| p.R918H | rs147357710 | 3828312 | 1.4*10^-4^ | 27.4 | 4.4*10^-5^ | 0/0 | 1/0 | 0/0 | 0/0 |
| p.S924A | rs781124953 | 3828295 | - | 28.0 | 4.4*10^-5^ | 0/0 | 1/0 | 0/0 | 0/0 |
| Total rare carriers |  |  |  |  |  | 70/0# | 100/0## | 3/0 | 1/0 |

# there are two individuals carrying two mutations. ## there are three carriers carrying two mutations. Het: heterozygotes; Ho: homozygotes
