# Supplementary material for: Sequencing reveals protective and pathogenic effects on development of diabetes of rare GLIS3 variants
Source: PLoS One. 2019 Aug 15;14(8):e0220805. doi: 10.1371/journal.pone.0220805 (PMC6695102; doi:10.1371/journal.pone.0220805)
Supplement: S2 Table — (DOCX) [file pone.0220805.s002.docx]

**S2 table**: The effect of the p.I28V variants among non-diabetic participants.

| **Trait** | **Non-carriers of p.I28V* (*n*=5,716)** | **Carriers of p.I28V**  **(*n*=10)** | **P-value** |
| --- | --- | --- | --- |
| **Non-diabetic individuals (*n*=5,726)** |  |  |  |
| n (men/women) | **2,809/2,907** | **4/6** | NA |
| Age (years) | 45.9 (7.90) | 46.7 (7.58) | 0.5 |
| BMI (kg/m^2^) | 26.0 (4.43) | 26.9 (6.16) | 0.2 |
| Waist/hip ratio | 0.85 (0.09) | 0.86 (0.09) | 0.5 |
| Glycated hemoglobin (HbA1c %) | 5.79 (0.40) | 5.81 (0.42) | 1.0 |
| Glycated hemoglobin (HbA1c mmol/mol) | 39.8 (4.35) | 40.0 (0.42) | 1.0 |
| HOMA-IR | 1.67 (1.14) | 1.52 (0.71) | 0.8 |
| Fasting plasma glucose (mmol/l) | 5.45 (0.51) | 5.17 (0.34) | 0.05 (0.046) |
| 30-min plasma glucose (mmol/l) | 8.56 (1.70) | 8.38 (1.02) | 0.8 |
| 2-h plasma glucose (mmol/l) | 5.94 (1.53) | 6.28 (1.57) | 0.6 |
| Fasting serum insulin (pmol/l) | 40.8 (26.5) | 39.5 (18.4) | 0.4 |
| 30-min serum insulin (pmol/l) | 290.9 (181.6) | 243.4 (99.1) | 1.0 |
| 2-h serum insulin (pmol/l) | 204.0 (194.1) | 250 (23.4) | 0.9 |
| 0-minserum C-peptide (pmol/l.min) | 580.5 (252.2) | 555.1 (187.4) | 0.6 |
| 30-min serum C-peptide (pmol/l.min) | 2002 (715.6) | 1992 (637.3) | 0.9 |
| 2-h serum C-peptide (pmol/l.min) | 2254 (976.6) | 2332 (885.3) | 1.0 |
| Insulinogenic Index | 29.7 (19.3) | 25.2 (14.5) | 0.3 |

*all non-diabetic individuals not carrying thep.I28V variant including individuals carrying other *GLIS3* variants.
